# Sensing the Heat Stress by Mammalian Cells

**DOI:** 10.1186/2046-1682-4-16

**Published:** 2011-08-11

**Authors:** Jordan Cates, Garrett C Graham, Natalie Omattage, Elizabeth Pavesich, Ian Setliff, Jack Shaw, Caitlin Lee Smith, Ovidiu Lipan

**Affiliations:** 1 Department of Physics, University of Richmond, 28 Westhampton Way, Richmond, VA 23173, USA

## Abstract

**Background:**

The heat-shock response network controls the adaptation and survival of the cell against environmental stress. This network is highly conserved and is connected with many other signaling pathways. A key element of the heat-shock network is the heat-shock transcription factor-1 (HSF), which is transiently activated by elevated temperatures. HSF translocates to the nucleus upon elevated temperatures, forming homotrimeric complexes. The HSF homotrimers bind to the heat shock element on the DNA and control the expression of the hsp70 gene. The Hsp70 proteins protect cells from thermal stress. Thermal stress causes the unfolding of proteins, perturbing thus the pathways under their control. By binding to these proteins, Hsp70 allows them to refold and prevents their aggregation. The modulation of the activity of the hsp70-promoter by the intensity of the input stress is thus critical for cell's survival. The promoter activity starts from a basal level and rapidly increases once the stress is applied, reaches a maximum level and attenuates slowely back to the basal level. This phenomenon is the hallmark of many experimental studies and of all computational network analysis.

**Results:**

The molecular construct used as a measure of the response to thermal stress is a Hsp70-GFP fusion gene transfected in Chinese hamster ovary (CHO) cells. The time profile of the GFP protein depends on the transient activity, Transient(t), of the heat shock system. The function Transient(t) depends on hsp70 promoter activity, transcriptional regulation and the translation initiation effects elicited by the heat stress. The GFP time profile is recorded using flow cytometry measurements, a technique that allows a quantitative measurement of the fluorescence of a large number of cells (10^4^). The GFP responses to one and two heat shocks were measured for 261 conditions of different temperatures and durations. We found that: (i) the response of the cell to two consecutive shocks (i.e., no recovery time in between shocks) depends on the order of the input shocks, that is the shocks do not commute; (ii) the responses may be classified as mild or severe, depending on the temperature level and the duration of the heat shock and (iii) the response is highly sensitive to small variations in temperature.

**Conclusions:**

We propose a mathematical model that maps temperature into the transient activity using experimental data that describes the time course of the response to input thermal stress. The model is built on thermotolerance without recovery time, sharp sensitivity to small variations in temperature and the existence of mild and severe classes of stress responses. The theoretical predictions are tested against experimental data using a series of double-shock inputs. The theoretical structure is represented by a sequence of three cascade processes that transform the input stress into the transient activity. The structure of the cascade is nonlinear-linear-nonlinear (NLN). The first nonlinear system (N) from the NLN structure represents the amplification of small changes in the environmental temperature; the linear system (L) represents the thermotolerance without recovery time, whereas the last system (N) represents the transition of the cell's response from a mild to a severe shock.

## Background

Living organisms need to sense the temperature of their surroundings. The heat-shock response network provides this sensing and controls the adaptation and survival of the cell. This network is a highly conserved genetic network that is connected with many other signaling pathways.

**Figure 1:**
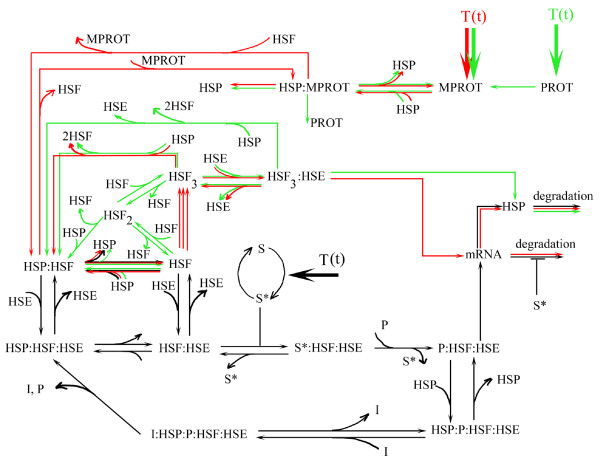
**Network models for the heat shock system**. The network from [2] is represented in black, from [3] in red and from [4] in green. HSP:HSF denotes HSP bound to HSF and similar for all other complexes. The solid lines represent chemical reactions and regulatory interactions.

Different aspects of this network were studied and different architectures were proposed in computationally oriented studies [1-4]. Our starting point was the network described in [2] because in that network architecture, temperature influences the hsp70 promoter activity directly. We were interested in the hsp70 promoter activity because our molecular construct is a Hsp70-GFP fusion gene transfected in Chinese hamster ovary (CHO) cells, as described in [5]. However, two other architectures from [3,4], use a different input for the temperature stress, an input that was proposed for the first time in a computational network by [1]. It is thus important to compare different heat shock network architectures. To this end and to connect our approach with the networks based on molecular interactions, we superimposed in Figure 1 three heat shock networks from [2-4], each network with a specific color and each molecular species named as in [2-4].

A key element in Figure 1 is the heat-shock transcription factor-1 HSF, which is transiently activated by elevated temperatures. HSF translocates to the nucleus upon elevated temperatures, forming homotrimeric complexes. The HSF_3 _homotrimers bind to the heat shock element HSE on the DNA and control the expression of the hsp70 gene. The Hsp70 proteins protect cells from thermal stress. Thermal stress causes the unfolding of proteins, perturbing thus the pathways under their control. By binding to these proteins, Hsp70 allows them to refold and prevents their aggregation.

Some interactions from Figure 1 are common to two networks, and are represented by lines with different colors. Only the binding and unbinding of the HSF to HSP70 and the HSP degradation appear in all three networks.

Although the networks share few common molecular species and have different architecture, they all share a common theme, namely they all have an entrance module that processes the input signal, which is the heat shock temperature as a function of time, T(t).

In [2] the temperature acts on a kinase module of Goldbeter and Koshland type [6]. The temperature changes the rate of conversion of the inactive kinase S into its active form S* Figure 1. The transition between the inactive and active form is modeled by(1)

where *S_tot _*= *S *+ S* is the total concentration of the kinase. The parameters *K_m, k_*, *K_m, p _*represent the Michaelis-Menten constants whereas *V_m, k _*and *V_m, p _*represent the maximal rates of phosphorylation and dephosphorylation of S*, respectively.

The ratio *V_m, k_*/*V_m, p _*controls the switching between inactive and active states of the kinase [6] and it was chosen in [2] as the variable that couples the heat shock network with the external environmental input temperature T(t)(2)

In [3,4] the temperature does not act through a kinase module, but directly on the proteins as a whole. No distinction was made between different families of proteins from the cells. The proteins were grouped in two classes: misfolded by the heat shock or correctly folded. Following [1] the fraction of the misfolded proteins, as a function of temperature, used in [3,4] is(3)

where, T(t) is in degree Celsius. In [3], the temperature influences the network through the rate of change of the misfolded proteins (MPROT in Figure(4)

whereas in [4] the temperature influences both the misfoled and the correctly folded (PROT in Figure 1) proteins(5)

A second common theme for all heat shock networks from [2-4] is an in depth study of the time variation of the hsp70 promoter activity. In [3,4] the promoter activity is controlled by HSF_3_:HSE, whereas in [2] by P:HSF:HSE. All three networks from Figure 1 incorporate the experimental findings of [7], that the promoter activity starts from a basal level and rapidly increases, once the stress is applied, reaches a maximum level and attenuates slowly back to the basal level. In [7] the stress was applied for 250 min at 42°*C *on HeLa cells. The rapid increase of the promoter activity spans tens of minutes (about 30 min in [7]) whereas the attenuation period extends over hundreds of minutes (about 200 min in [7]). This phenomenon, referred as transient activity, is the hallmark of all three network studies [2-4]. In [2,3] the hsp70 mRNA has a transient activity also, which is regulated by S* in [2].

The transient activity is experimentally probed in [4] with the help of a yellow fluorescent protein YFP as a reporter. The transactivation of the yfp gene is controlled by its own HSE' elements. In [4] the connection between the reporter rate of accumulation and the HSF_3_:HSE' is(6)

The constant *k*_1 _describes the transcription/translation kinetics, [4], whereas *k*_2 _describes the degradation of the YFP protein. Using the networks from [2,3] we write the YFP accumulation rate as a function of the yfp-mRNA(7)

In all cases considered, based on the network models, the rate of change of the reporter protein has a transient activity. We can write thus, in general, that(8)

Following [2,3], Transient(t) depends on mRNA and its controlled regulation, as we discussed in (7). Following [8], we may argue that Transient(t) depends strongly on the translation initiation effects elicited by the heat stress.

The architecture of the heat shock network will change, as more experimental data will accumulate, but the transient activity of the rate of accumulation of the reporter controlled by the hsp70 promoter will still be present. What will change is the dependance of the Transient(t) function on specific molecular species. For example, the transcriptional corepressor CoREST, which may be important to be included in any new heat shock system network architecture, may influence Transient(t) [9].

The molecular construct we used to measure the response to thermal stress is a Hsp70-GFP fusion gene transfected in Chinese hamster ovary (CHO) cells, as described in [5]. The time profile of the GFP protein is recorded using flow cytometry measurements, a technique that allows a quantitative measurement of the fluorescence of a large number of cells (10^4^).

Considering a similar model as in (8) for our GFP reporter, we get(9)

To obtain a mathematical representation for Transient(t), we will use the following parametrization of the GFP response(10)

**Figure 2:**
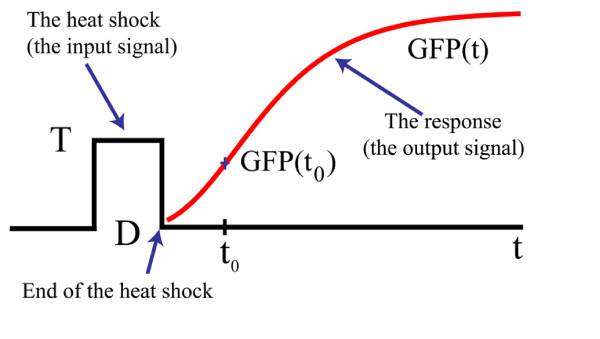
**The response to a heat shock pulse**. This GFP reporter response is measured after the heat shock is over. The profile of the response depends on how the cell records the stress during the shock. This profile gives an insight into cell's processes that transforms the shock into a molecular activity.

This form for response was also used in [5] to study the response to one heat shock pulse of a definite temperature T and duration D, Figure 2. This is not the only possible parametrization for the GFP accumulation. Some parametrization may be based on the Michaelis-Menten approach, including a Hill coefficient. However, (10) proved very useful in constructing a stochastic model for the GFP accumulation in [5] and it is easy to use it to model a transient activity.

The parameters *a *and *b *in (10) depend on the input shock. The GFP(*t*_0_) is the value of the GFP measured at some time *t*_0 _after the end of the shock. From a theoretical point of view, the time *t*_0 _can be any time after the end of the shock. Practically though, the time *t*_0 _must be chosen so that the interval between the end of the shock and *t*_0 _is not very long compared with 24 hours. After the shock, to measure GFP(t), samples were taken for the next 24 hours at a rate of one sample at every two hours. We sampled, simultaneously, 13 different shock conditions, for the next 24 hours. The delivered shocks, for different conditions, did not end at the same time. As a consequence the time *t*_0 _for the first sample was different for different conditions. The experimental values for *t*_0 _covered a range from 0.5 hours to 1.8 hours.

The expression (10) is valid only after the end of the heat shock and covers a time range of about 24 hours.

With the help of (10) and (9) the function Transient(t), measured after the end of the shock to the next 24 hours, is expressed in the form(11)

From now on the transient activity and Transient(t) defined above will be used interchangeably. This gives a parametrization for the transient activity in terms of two parameters *a *and *b*. Based on the discussions related to (6,7), the parameters *a *and *b *depend on molecular species that are part of a yet unknown heat shock network.

The temperature dependence of the Transient(t) is therefore a combination of different pathways and feedback loops. A detailed understanding of the entire network responsible for the heat-shock response will make it possible to deduce the temperature dependance of the Transient(t). Because a detailed understanding of the network is missing at present, our approach will be top-down. Namely, our goal is to obtain Transient(t) from experimental data that describe the time course of the GFP response to input thermal stress.

In other words, instead of looking at HSF_3_:HSE' or mRNA or other molecular species of an incompletely defined heat shock network, as the output signal of the heat shock system, we consider *a *and *b*, that describe the transition activity, as the output of the heat shock system. Our procedure does not imply that searching for a mechanistic architecture for the heat shock system is futile. Contrary, we believe that it is worthy of finding the parameters *a *and *b *in terms of concrete molecular species. However, this is not the purpose of this study. Out of the two parameters *a *and *b*, we will focus on *b *because it behaves like a time constant in (11) and thus describes the Transient(t) life time as a function of the input temperature.

**Figure 3:**
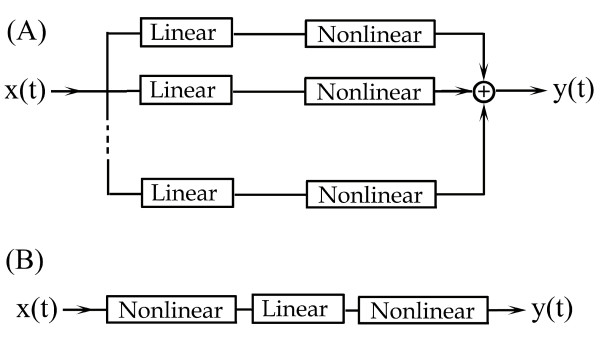
**The parallel-cascade nonlinear model**. (A)The branches consist of a linear time-invariant system connected in series with a memoryless nonlinear block. (B) The Nonlinear-Linear-Nonlinear model for the heat shock system.

To find the mathematical model for b as a function of the input temperature, we will be guided by the parallel-cascade system from Figure 3.

In many applications, nonlinear systems are described using simplified structures, [10,11] and reference therein. The structure of Figure 3A is composed of parallel branches, with each branch represented by a series connection of a linear time-invariant system (L) connected with a memoryless nonlinear system (N). The transfer function for the linear system (L) is described by a convolution integral [12], whereas the nonlinear memoryless system (N) is described by a nonlinear function *f*(*t*). The output *y*(*t*) of the LN parallel-cascade system of Figure 3A is(12)

where the summation index *i *runs over all branches. For each branch, the function *g_i_*(*τ*) rep-resents the linear system and *f_i_*(*τ*) the nonlinear system, respectively. The importance of this setting is that a large class of nonlinear systems can be uniformly approximated using a parallel combination of several L and N cascade systems [10]. The structure of Figure 3 is not the only possible structure. For example, [13] uses for each branch three systems, linear-nonlinear-linear, so the parallel system is an LNL cascade. As an example of a general theorem that describes the uniform approximation of nonlinear systems using L and N structures, we state, without proof, the theorem of Palm [13]. A time-invariant, causal, finite-memory system for which small changes in the the system input result in small changes in the system output, can be uniformly approximated by a parallel-cascade system formed with a finite number of branches, each of which contains an LNL cascade, [11,13].

For the present study, we will use a single branch with an NLN structure, Figure 3B. We chose the NLN structure because each simple system represents a process that is important for the heat shock response. The first nonlinear system (N) from the NLN structure represents the amplification of the small changes in the environmental temperature. Similarly with (2,4,5), the functional task of the first module N from our cascade model is to processes the heat shock temperature T(t).

The linear system (L) represents the thermotolerance without a recovery period. Thermotolerance is the ability of cells to better tolerate a strong second heat shock once previously exposed to a first moderate shock [14]. An entire subsection is devoted below to the phenomenon of thermotolerance without a recovery period and its connection to the linear system (L). The last system (N) represents the transition of the cell's response from a mild to a severe shock. We will explain each block and its corresponding function in the next sections.

In general, the identification of a nonlinear system using a parallel-cascade structure does not require its N and L systems to bear resemblance to the physical nature of the modeled system. For our study, however, we ask for resemblance because we view the model structure in connection with a molecular heat shock network.

The kernel functions *f*(.) and *g*(.) that describe the nonlinear and linear blocks, respectively, are usually represented as polynomial, rational or exponential functions. We will use exponential functions to construct the kernels of the N and L systems. One reason for using exponential functions is that the amplification (the first N system in the NLN casacde) and the thermotolerance without recovery time (the linear system L) become very simple kernel functions, namely one exponential for the first N system and another one for the L system. Another reason is that we found experimentally that small temperature variations largely influences the response output of the heat shock network. Amplification of small variations is conveniently described by exponential functions. We notice also that in (3) the temperature enters through an exponential function, which expresses, as we mentioned, the strong sensitivity of the heat shock system to a 1°*C *temperature change. A strong temperature dependence is also present in [2], where the ratio *V_m, k_*/*V_m, p _*changes five orders of magnitudes for a temperature change of 4°*C*.

For consistency, the last N system in the NLN cascade will be modeled also using an exponential function, although it must be multiplied by a linear function to express the transition from mild to severe stress response. As a general approach, we also aimed to use as few parameters as possible to construct each of the kernel functions of the NLN cascade.

Below we explain the building of the NLN model out of experimental data, together with the logic we used at each step and the tests we applied to check the modeling process.

We stressed the CHO cells with different heat shocks profiles. For linear systems, and from a theoretical point of view, it will suffice to use a Dirac-*δ *input. However, we cannot impose too high of a temperature on cells. So, we need to use a set of one pulse shocks, (*T*, *D*) of variable temperature *T *and duration *D*. The goal is thus to find *b*(*T*, *D*) as a function of *T *and *D*.

Because the system is nonlinear, the response to one shock will not reveal its nature. For this reason we extend the set of input shocks to contain also double shocks of different temperature levels and durations. For a double shock input, (*T*_1_, *D*_1_) followed by (*T*_2_, *D*_2_), the *b *will depend on all four variables, *b*(*T*_1_, *T*_2_, *D*_1_, *D*_2_). We will only study the case for which the time between the shocks is negligible, a point that will be elaborated in more detail in connection with the definition of thermotolerance. For all double shocks, the time between shocks was about two minutes which was necessary for replacing the media used for the first shock temperature with the media for the second shock temperature. The replacement included a centrifugal spinning at the end of the first shock, followed by cell resuspension into the second shock media. The shocks from a two shock sequence are thus distinct, with a short time interval in between. Details on shock delivery are described in Material and Methods. The reason that *b *is seen either as a function of two, (*T*, *D*) or four,(*T*_1_, *T*_2_, *D*_1_, *D*_2_), variables is because we regard *b *as a function of the input heat shock, *b*(Heat Shock). The *b*(*T*, *D*) and *b*(*T*_1_, *T*_2_, *D*_1_, *D*_2_) functions appear as a result of the composition of the function *b*(Heat Shock) with the function Heat Shock(*T*, *D*) and, Heat Shock(*T*_1_, *T*_2_, *D*_1_, *D*_2_), respectively.

If we can find the function *C*(*x*, *y*) that describes the composition law *b*(*T*_1_, *T*_2_, *D*_1_, *D*_2_) = *C*(*b*(*T*_1_, *D*_1_), *b*(*T*_2_, *D*_2_)), we can find the cell's response to three or more adjacent shocks, under the natural hypothesis that the composition law is associative. We thus limit our input stresses to single and double shocks. In what follows we explain a series of 261 experimental conditions and the theoretical reasonings that led us to propose a model for the transient activity (the b-values) as a function of the input stress. The question about the mathematical model that describes the function *b*(*T*, *D*) was left open in [5]. The only reference about *b*(*T*, *D*) was Figure 9B in [5], which is corrected in the present study by removing the saddle point. The improvement is due to an increase in the number and the structure of the shocks from 48 in [5] to 261 in the present study.

## Results

### One shock and mild to severe stress transition

Previous studies recognize two classes of stress: mild and severe, [5,15,16]. A severe heat shock leads to a transient arrest of the cell cycle mainly at two check-points, the G1/S and G2/M transitions. The cell also may enter apoptosis after a severe heat shock. Contrary to a severe stress, a mild stress is common for physiological conditions. During febrile diseases, the body temperature usually increases by 1 - 2°*C*. There is no precise criteria to separate a mild from a severe shock. A broad classification places a heat stress in the temperature range 39 - 42°*C *for a duration of 15 - 20 minutes in the mild category, whereas a shock in the temperature range of 43 - 45°*C *for the same durations interval will be considered severe [16].

**Figure 4:**
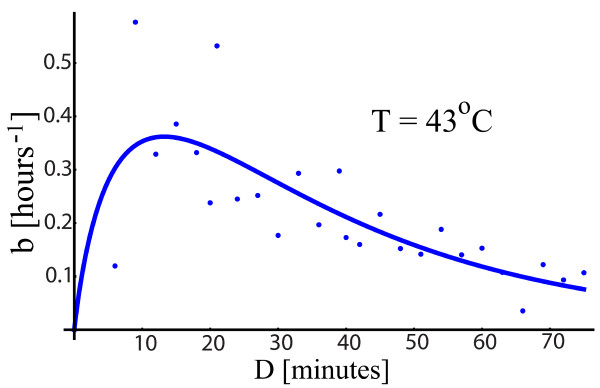
**Mild to severe transition**. As the duration of the heat shock extends, the b-values increase up to a maximum value from which it gradually decreases down to low levels.

**Figure 5:**
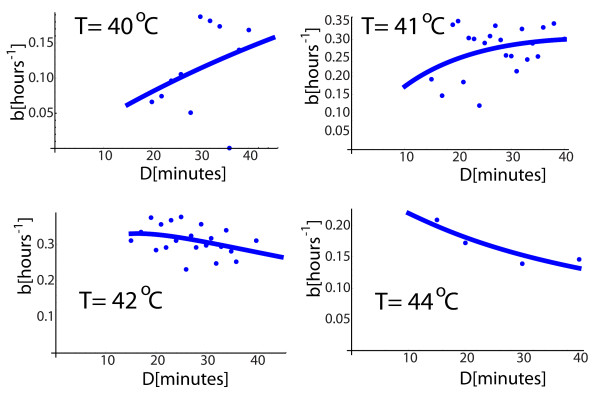
**Mild an severe responses at different temperatures**. At *T *= 40°*C *and *T *= 41°*C *the cell is under a mild stress and the activity is on the rise. For *T *= 42°*C *the stress is transitioning from mild to severe whereas at *T *= 44°*C *it is extremely severe.

To search for a mild to severe transition in the response, we kept the temperature constant and gradually increased the duration of one heat shock pulse. Because 43°*C *is a borderline temperature we see such a transition as we increase the heat shock duration from 6 to 75 minutes by an increment of 3 minutes. The *b *parameter increases up to about 15 minutes when it reaches a maximum. After that, for longer durations, *b *gradually declines towards lower values, Figure 4. We say that, for durations from *D *= 0 up to the duration when *b *achieves its maximum value, the shock (*T*, *D*) is mild, otherwise being severe. Similar results were obtained for other temperature, for which the activity was measured for shock durations in the time interval 15-40 minutes Figure 5. At *T *= 40°*C *and *T *= 41°*C *the cell is under a mild stress and the activity is on the rise. For *T *= 42°*C *the stress is transitioning from mild to severe whereas at *T *= 44°*C *it is extremely severe. At any temperature, there is thus a duration *D_max _*for which the activity reaches a maximum level. For durations from zero to *D_max _*the activity increases and it declines for durations longer than *D_max_*. The transition duration *D_max _*depends on temperature, *D_max_*(*T*), and is higher for lower temperatures. For *T *= 41°*C*, *D_max_*(41) is greater than 40 minutes; for *T *= 42°*C*, *D_max_*(42) is about 20 minutes; for *T *= 43°*C*, *D_max_*(43) is just above 10 minutes whereas for *T *= 44°*C D_max_*(44) is below 10 minutes, Figures 3 and 4. This behavior is not surprising, because we expect the same response for low temperature and higher duration as for higher temperature and shorter durations. The simplest idea would be that for the same response the product between temperature elevation and duration, (*T *- 37)*D*, is the same. However, based on the experimental results presented above, the requirement to keep the product (*T *- 37)*D *constant to get the same response is a coarse approximation. This approximation will predict that *D_max_*(41)/*D_max_*(44) is 7/4 whereas the data show that it is closer to 10. Before we remove this coarse approximation from the model, it will be used as a guiding procedure to express the transition profile of Figure 4 as a function of the temperature and duration of the heat shock.

To describe the profile of Figure 4, we search for a function that continuously transits from an increasing behavior for mild shocks to a decreasing regime for severe shocks. The precise mathematical form of the function is not critical; its purpose is to represent the mild-severe transition using few parameters. As explained in the introduction, the class of functions that we will use to parametrize the mild-severe transition will be based on an exponential function:(13)

The part *Ax^n ^*describes the effect of a mild stress, whereas  is responsible for the response to a severe stress. This class of functions was used to fit the data in Figures 4 and 5 with a smooth curve. To keep the model simple, the response to a mild regime is considered to be linear, so we took *n *= 1.

To have a temperature-dependent profile that captures the experimental data we take *x *= (*T *- 37)*D *and get *b*(*T*, *D*) = *f*((*T *- 37)*D*) keeping *A*, *n*, *λ *and *m *temperature-independent. The response *f*((*T *- 37)*D*) will be the same if (*T *- 37)*D *is kept constant, so we recovered the coarse approximation from above. The response reaches its maximum for a shock of duration *D_max _*= *x_max_*/(*T *- 37) where *x_max _*is the argument for which *f*(*x*) is maximum. The value *x_max _*depends only on *A*, *λ *and *m *and is thus temperature-independent. So *D_max _*decreases as temperature increases, in qualitative agreement with the experiment.

In what follows, as the NLN model improves, the simple substitution *x *= (*T *- 37)*D *in *f*(*x*) to get *b*(*T*, *D*) will be modified. This simple substitution will not be completely rebuked by the improved NLN model because both coincide for very mild shocks.

### Thermotolerance with no recovery time, double shocks and the convolution integral

Thermotolerance is the ability of cells to better tolerate a strong second heat shock once previously exposed to a first moderate shock [14]. Thermotolerance is usually studied by inserting a long period of time (hours) between the first and the second heat shock [17]. The first shock is viewed as a pre-conditioning stress, the assumption being that thermotolerance is due to the heat shock proteins which act as chaperones to prevent protein aggregation during the second heat shock. Thus, to acquire thermotolerance the cells need a period of recovery to make the heat shock proteins. Recovery periods of 3, 6, 12 and 24 hours, followed by a second shock were used in [17]. In the same study the response was measured at 1 hour after the second shock.

From a computational point of view, the study [3], remarks that, subsequent to the heat shock, more heat shock proteins are produced inside the cell. Since these proteins are very stable they act in an anti-apoptotic manner for a longer period of time and therefore they are responsible for the cell's adaptation for the next stress.

Viewed through this experimental scenario, the definition of thermotolerance must include a non-negligible recovery period between the shocks. However, the experimental definition of thermotolerance may be enlarged to include a negligible recovery period, keeping the theoretical requirement of [14] that the response to the second shock be dependent on the intensity of the first shock. In other words, the response of the cell to two shocks of different temperatures depends on the order of the input shocks, that is two shocks of different temperatures do not commute. Thermotolerance with negligible recovery time may be generated by different sources, like HSF DNA binding activity, post-translational modification of HSF, mRNA stability [2] and accumulation of the end products of HSF activation. It is not the purpose of this paper to study the biochemical origin of the thermotolerance without recovery time. Such a study will be very interesting, especially to find the differences in the biological pathways that are responsible for thermotolerance with and without recovery time.

From know on, when we refer to thermotolerance we imply that the recovery time is negligible in comparison with the shocks' durations. In what follows when we refer to the noncommutativity property for a pair of shocks, we understand that noncomuttativity implies thermotolreance and not viceversa. For example two identical shocks commute but the thermotolerance is still present. The response to the second shock is influenced by the presence of the first, identical, shock.

**Figure 6:**
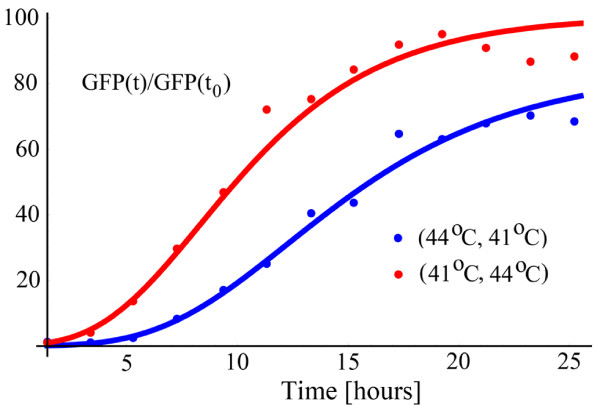
**Thermotolerance with no recovery period as a noncommutativity property**. The reference point is *t*_0 _= 1.43 hours for the (41, 44) pair, and *t*_0 _= 3.67 hours for the (44, 41) pair.

The thermotolerance, expressed as the noncommutativity of the shocks, is evident in Figure 6 where the responses for (41°*C*, 44°*C*) and (44°*C*, 41°*C*) are displayed. The response is slower and delayed when the stronger shock is applied first. For this reason, the reference time of *t*_0 _= 3.67 hours for the (44°*C*, 41°*C*) pair is taken at a later time than *t*_0 _= 1.43 hours for the (41°*C*, 44°*C*) pair. Each shock used to produce the responses in Figure 6 has a duration *D *= 20 minutes.

**Figure 7:**
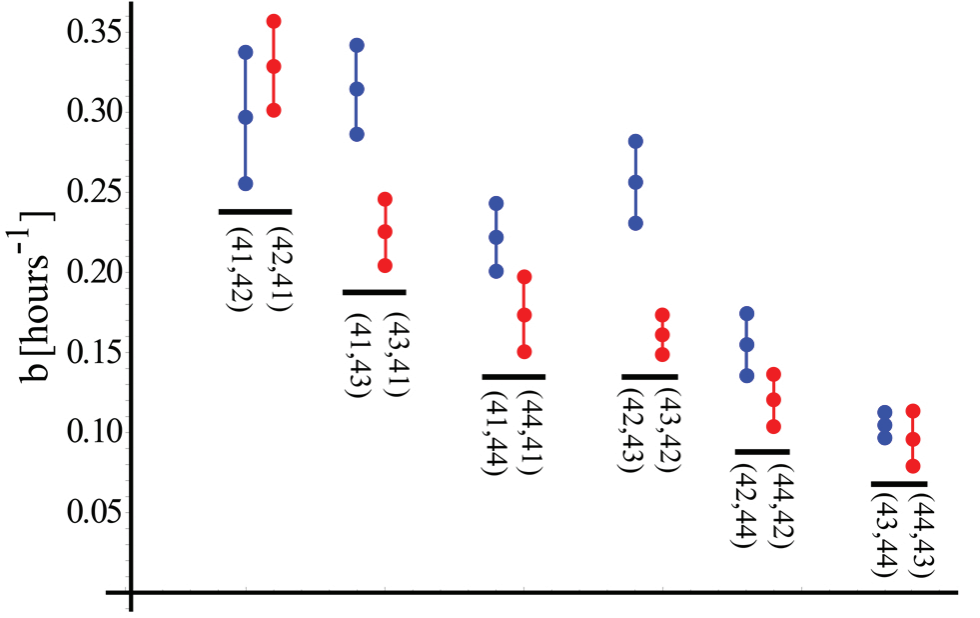
**The noncommutativity in the transient activity**. In each vertical triplet, the middle point represents the estimated value for *b*, whereas the upper and the lower point represent the 95% confidence bounds. The triplet points are grouped in pairs.

In Figure 7, each vertical triplet of points refers to the *b *parameter obtained from twelve different conditions. The mid-point of a vertical triplet is the estimated *b*, whereas the upper and the lower points represent the 95% confidence bounds. Pairs of temperatures shocks, each shock being delivered for 20 minutes, are shown below the triplets. The noncommutativity of shocks is evident, except for the conditions (43°*C*, 44°*C*) and (44°*C*, 43°*C*). For these conditions, the temperatures are high and the cell starts to respond differently than when the input shocks are mild. This mild versus strong behavior will play an important role as we develop the model. For now, however, we will concentrate on a simple mathematical mechanism that describes the composition rule for the response under a sequence of two or more pulses.

To that end, consider that the heat shock temperatures are just above 37°*C *and the durations are small(few minutes or less). If two shocks are delivered, in the order (*T*_1_, *D*_1_) followed by (*T*_2_, *D*_2_), we propose to describe the b-value for this very mild double shock as a weighted combination of the input temperature shocks(14)

The proposed combination is linear because at this point we consider heat shocks close to 37°*C *and for short durations. In the absence of thermotolerance, the weights should be equal. However, because of the thermotolerance, the first shock weights more than the second, *w*_1 _>*w*_2_. The weights *w*_1_, *w*_2_, depend on the durations *D*_1_, *D*_2_. The linear approximation (14) for a very weak heat stress is supported by the findings of [3]. In this study it was found that when the temperature is slowly increased from 37°*C *to 42°*C *over a duration of 5 hours, the transient activity just barely raises above the basal level. This "slow heat" effect, as it was called in [3], supports thus our linearity assumption for the response for weak temperature inputs. Because we are studying only thermotolerance without recovery periods, for a sequence of *k *pulses, each of short duration, and all adjacent to each other, the b-value will be given by(15)

Thermotolerance implies that the weights will decrease from one pulse to the next(16)

The linearity hypothesis for a sequence of pulses is challenged because with the accumulations of many shocks, the cell will transit into a nonlinear response regime. We will include later the nonlinear effects, exploring for now only the linear effects. The form (15) points immediately to the well-known response of a linear, time-invariant system, as a convolution sum(17)

with *w_m _*= *g*(*k *- *m*). A continuous input heat shock is described by a time-dependent temperature input *T*(*t*). The continuous version will thus be described by the convolution integral(18)

The shock starts at *t *= 0 and continues afterwards. At every instant of time *t*, the cell respond to the incoming stress and the transient activity changes as the time *t *progresses. This activity depends on the entire history of previous shocks, which is expressed by the integral. The thermotolerance is described by the weight function *w*(*τ*) = *g*(*t *- *τ*), which must be a decreasing function of *τ *for fixed *t*. As explained in the introduction, we will use an exponential function to build the NLN cascade systems; thus take *g*(*t *- *τ*) = *Be*^*β*(*t *- *τ*)^, with *B *> 0, *β *> 0.

The thermotolerance is now expressed by the requirement that *β *be a positive number. We emphasize here again that the model is not a representation of a detailed molecular network. It is a model that represents the measurements and describes a reduced equivalent system of a yet unknown detailed network structure.

We thus express the b-values to a continuous temperature variation *T*(*τ*), in the mild regime, as the convolution integral(19)

It is known that a convolution integral is the simplest expression of a response that encapsulates system's memory, [12]. The cell's response at present time, *t*, is influenced by the entire memory accumulated from previously imposed shocks, *T*(*τ*) - 37 with 0 ≤ *τ *≤ *t*.

For one shock at temperature *T *and for a duration *D*, the prediction of (19) is a b function of the form(20)

This implies again that a short duration pulse at high temperature will induce the same response as a longer pulse at a lower temperature.

For small durations *D*, the activity for one shock becomes *b*(*T*, *D*) = *BD*(*T *- 37) and we recover the coarse approximation which connects constant responses by the requirement *D*(*T *- 37) = *constant*. From (20), the improved condition for constant response is (*e*^*βD *^- 1)(*T *- 37) = *constant*. We check this new condition against the experimental data. For the pairs (*T*_1_, *D*_1_) = (41, 40) and (*T*_2_, *D*_2_) = (43, 15) the responses are similar, Figures 4 and 5. However, there is no positive *β *for which  because , as a function of *β*, increases above its zero value for *β *= 0. Its derivative with respect to *β *is positive because, for all positive *β*(21)

This inequality gives an insight on finding a cure for the lack of agreement with experiment. The inequality (21) is valid because the duration *D*_1 _is much larger than *D*_2 _while, at the same time, the temperature elevations *T*_1 _- 37 and *T*_2 _- 37 are not so different. To break down the inequality (21), it is necessary for the temperature to appear not simply as a difference from the basal 37°*C*, but to appear in such a form for which an increase of 1°*C *has a greater impact. The requirement agrees with the common observation that an increase from 37°*C *to 38°*C *in body or tissue temperature affect its functionality.

Again, we are going to use an exponential function to detect small changes in temperature and correlate large variations in duration with small variations in temperature:(22)

This exponential expression was chosen so that for temperatures close to 37°*C *it reproduces the above *T*(*t*) - 37 linear dependence. It is worth to mention again that in (3) the temperature enters through an exponential function as well.

Using the exponential function for the temperature, the b function (19) turns into(23)

**Figure 8:**
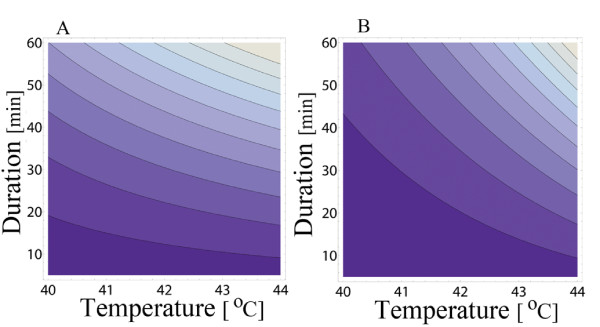
**Constant contours for the transient activity**. For one shock of duration D and temperature T, the b-values are constant along hyperbolic-like curves. One pulse of short duration and high temperature induces the same b-value as a pulse of longer duration but smaller temperature. (A) The contours are less steep for a linear detection *T *- 37, than (B), for an exponential detection *e*^*α*(*T *- 37) ^- 1. Here *β *= 0.02min^-1 ^and *α *= 0.4°*C*^-1^.

The response to one shock becomes(24)

and it is possible now to find two positive constants *α *and *β *so that the pairs (*T*_1_, *D*_1_) = (41, 40) and (*T*_2_, *D*_2_) = (43, 15) give the same response. The constant activity contours,(25)

drop more steeply for an exponential temperature detection, *e*^*α*(*T *- 37) ^- 1 for Figure 8B, than for the linear temperature detection, *T *- 37 for Figure 8A. The contour plots for the parameter b described in Figure 9B in [5] are now replaced by Figure 8B, where the saddle point is absent. This conclusion does not affect the results of [5] which are independent of the saddle point.

**Figure 9:**
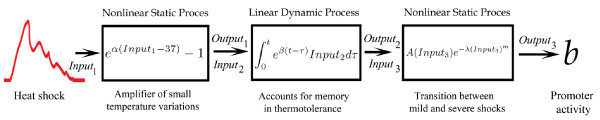
**The block diagram for the transient activity**. The environmental temperature, *Input*_1_, passes through three blocks to regulate the transient activity. The cascade connection implies that the output signal from one block equals the input signal for the next block. The time intervals on which *Input*_1 _is different from 37°*C *are adjacent to each other (no recovery time).

There is one more piece of information to add to the model, namely the nonlinear effect of a severe stress. The nonlinear transition from a mild to a severe response was obtained in the previous section by using the composition *f*(*x*(*T*, *D*)) with *x*(*T*, *D*) = *D*(*T *- 37), (13). The coarse grain approximation for constant response condition *D*(*T *- 37) = *constant *is now replaced by (25). This improved condition suggests using *x*(*T*, *D*) = (*e^αD ^*- 1)(*e*^*α*(*T *- 37) ^- 1) in *f*(*x*) of (13) to get the transition from mild to severe stress response. This suggestion can be pushed forward if the convolution integral response is used in its most general form (23), and not only in its special form as it takes for one shock (24).

With this last inductive reasoning, the model for the b function achieves it final form. The response to a general time-dependent temperature *T*(*τ*) is(26)

with *f*(*x*) of the form(27)

The constant *B *in (24) was absorbed in the constants *A *and *λ *of the function *f*(*x*).

For small values of *x *the function *f*(*x*) becomes linear, *f*(*x*) ≈ *Ax*, and, as expected, the general expression (26) goes into the expression valid for mild stresses (23). For a severe stress, the response is described by the decreasing tail of *f*(*x*).

The expression (26) is represented as a series connection of three functional blocks in Figure 9. The input into the first block is the heat shock as a function of time, *T*(*τ*). The first block accounts for the sensitivity of the temperature detector because variations above the 37°*C *are amplified by the exponential sensor. The output of the first block becomes the input of the middle block which encapsulates the memory of the system (thermotolerance). The output of the middle block turns into the input of the last block, which describes the cell's transition from a mild to a severe response. The block diagram represents a NLN cascade model, as described in the introduction. The model for the heat shock detection is simple in the sense that it does not have parallel branches; it consists of only one branch. The three blocks connected in cascade are of the general type mentioned above: dynamic linear and static nonlinear. It is useful to comment again on the fact that (26) is valid for an input temperature function *T*(*t*) that is not zero on a duration range of about an hour, and that the b-value represents the transition activity from the end of the temperature stress up to about 24 hours.

In what follows we check the model against experimental data.

### Experimental results linked by the shared theoretical model

As mentioned in the introduction, the experimental results are based on one and two shocks, respectively. All experiments are summarized in the Material and Method section. For each input heat shock, and for each sampled time, the raw data is represented by at least ten thousand measured GFP intensities. For this reason, the raw data is not presented in print, being available in electronic format, from authors, by request. However, the raw experimental data were used to estimate the b-values, and these experimental results are presented in the manuscript's figures.

We will use the one shock experiments to estimate the parameters *α*, *β*, *A*, *λ *and *m *of the theoretical model (26) or Figure 9. Then the theoretical model is tested against the two shocks experiments. It is true that we used the thermotolerance with no recovery period, i.e. the general idea about the two shocks noncommutativity, to establish the convolution integral block in Figure 9. However, specific experimental results from the two shocks experiments were not used to set up the theory. These experimental details from the two shocks inputs will be tested against the theoretical model.

To this end, we can fit the model (26) using the 135 experimental one-shock conditions (see Materials and Methods) to estimate five parameters *A*, *λ*, *m*, *α *and *β*. This kind of fitting is equivalent to a an optimization problem in a 5-dimensional space, searching for a global minimum of a cost function. However, the present study is not about fitting experimental data to a proposed model. It is about the block structure of the model and its connection to different experimental results. For this reason, we will avoid using the 5-dimensional space and will take advantage of the composition property represented by the last block of Figure 9. The structure of Figure 9 allows us to use the first two blocks to compute a score for the shock, which is subsequently transformed by the last block into the b-value. The meaning of the score in this context is to evaluate and assign a grade to the stress, and not as a measure of the distance between experiment and theory, as is used in estimation theory.

For one shock at temperature *T *and duration *D *the score is (*e^βD ^*- 1)(*e*^*α*(*T *- 37) ^- 1), the same expression that appears in (25). To see this, from (26), we get(28)

were , and , . The b-value for one shock is thus given by the composition of a function  from the same class as the function *f*(*x*), (13), with the score (*e^βD ^*- 1)(*e*^*α*(*T *- 37) ^- 1). The advantage of this point of view is that the 5-dimensional space is split in a 2-dimensional space of the parameters (*α*, *β*) and a 3-dimensional space of .

For given (*α*, *β*), the score can be computed for all 135 one-shock conditions. The plot of the experimental values for *b *versus the computed scores will reveal the shape of the function . This shape is then easy to find through an optimization problem in the 3-dimensional space of . The question remains on how to find (*α*, *β*). Varying (*α*, *β*), the scatter plot of the experimental *b *values versus the *score *becomes broken, with islands of data points grouped together. For some values of (*α*, *β*) the islands disappear and the scatter plot starts to resemble a continuous function. We are looking thus for those *α *and *β *for which the scatter plot of *b *versus *score *becomes as close as possible to a continuous curve. For the continuity criteria we will use the variation  computed over the whole experimental scores (*score*_0_, *score*_1_, · · ·). With this criteria, the chosen (*α*, *β*) pair is that one for which the variation attains its minimum. We searched for (*α*, *β*) in discrete steps of 1/100 starting from (0, 0) and find that a minimum is achieved for values of *α *< 1 and *β *< 0.1. We get *α *= 0.63°*C*^-1 ^and *β *= 0.026 min^-1^, by searching the interval (0, 0.1) in steps of 1/1000. For values of *α *> 1 or *β *> 0.1, the scatter plot of the experimental *b *values versus the *score *does not resemble a continuous function, so we did not search for another potential minimum in this region.

**Figure 10:**
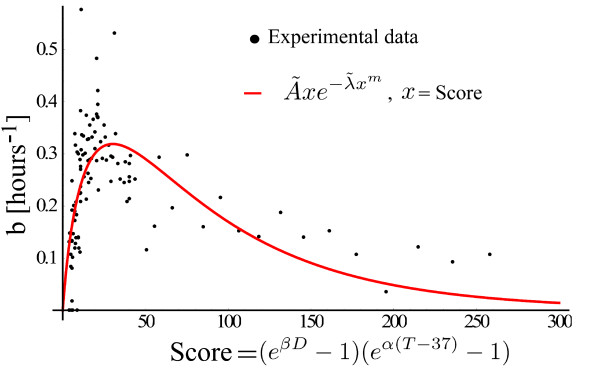
**Transient activity as a function of the stress' score from one-shock experiments**. The parameters *α *= 0.63°*C*^-1 ^and *β *= 0.026 min^-1 ^minimize the variation of the experimental b-values versus the stress' score. The fitted curve has the parameters , , *m *= 0.67.

The scatter plot was then fitted with the one-dimensional function  of three variables, which produced the values  hours^-1^, , and *m *= 0.67. The results are plotted in Figure 10 where 113 one-shock experiments were used. The values *α *= 0.63°*C*^-1^, *β *= 0.026 min^-1^,  hour^-1^, , and *m *= 0.67 can now be used to compute the theoretical responses to the double shocks experiments. The one-shock Experiment 7 from Materials and Methods was not used to estimate the parameters. For a 39°*C *shock the GFP level is very low, so a good estimation of the b-value is not attainable. However, this experiment supports the exponential sensitivity for temperature detection, because an increase of 1°*C *produces detectable information in the GFP levels.

**Figure 11:**
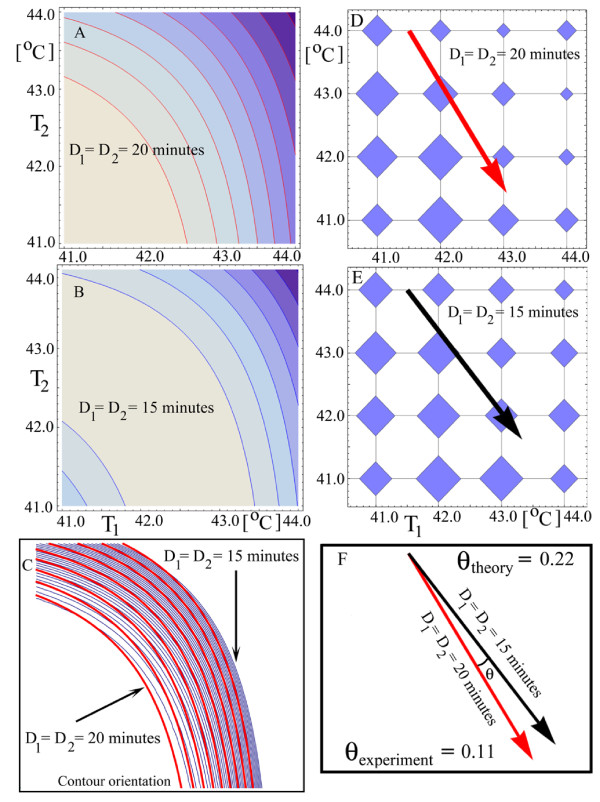
**Theoretical contour plots for two-shock experiments of equal duration but different temperatures (left column). **The b-values represented as discrete weights placed on a grid (right column). The orientation of the constant levels curves (A) and (B) depends on the shocks' duration. The contours for *D*_1 _= *D*_2 _= 15 minutes are rotated anticlockwise with respect to the contours for *D*_1 _= *D*_2 _= 20 minutes, (C). Each diamond in (D) and (E) represents a weight proportional with the b-value corresponding to the experimental condition to which the diamond is attached. For each figure, the arrow represents the direction of one principal axis, the other principal axis being perpendicular to it. The anticlockwise rotation predicted by the theory is confirmed by the experimental angle, (F).

With the five parameters estimated, we turn to compare the theoretical predictions with the experimental data for the two-shocks experiments. The experimental values for the parameter b for two-shocks experiments depend on four experimental variables, two temperatures and two durations, *b*(*T*_1_, *D*_1_, *T*_2_, *D*_2_). During experiments, two variables were kept constant, whereas the other two, call them (x, y), were varied, making the b-values a function of two variables, *b*(*x*, *y*). Viewed in a 3-dimensional space (*b*, *x*, *y*), the 2-dimensional surface represented by b(x, y) has a shape and position that depends on the specific combinations of the *T*_1_, *D*_1_, *T*_2_, *D*_2 _values of the input temperature. We will compare the surface *b*(*x*, *y*) obtained from the experimental values with the one predicted by the model, both generated by the same input temperature. The precision of this comparison grows with the number of discrete points (x, y) that represent the experimental *b*(*x*, *y*) surface. We were able to parallel measure up to 32 different input stresses using one cell batch, so the number of (x, y) achievable points is between 16 and 25. To increase the number of inputs into thousands, it is necessary to use an automated robotic system. This leap in the number of inputs will show subtle structures in the *b*(*x*, *y*) surfaces. In this way, local properties of these surfaces, measured experimentally, may be checked against the theory. Lacking an automated robotic system, we are bound to study the experimental *b*(*x*, *y*) surfaces estimated on 16 or so (x, y) pairs. Although local properties of the experimental *b*(*x*, *y*) surfaces cannot be studied, we can study global properties of these surfaces and compare them with the theoretical model. The global properties that we are going to study are best visualized by looking at the *b*(*x*, *y*) surface projected on the (*x*, *y*) 2-dimensional plane, with the values *b*(*x*, *y*) attached to each point (*x*, *y*). To get a global property, we borrow tools developed in mechanics, and consider that the value *b*(*x*, *y*) attached to each point (*x*, *y*) is the value of a point mass at that location. In this way, the (x, y) experimental space becomes a rigid body with point masses given by the experimental b-values, Figures 11D and 11E where each diamond represents a weight proportional to the measured b-value. The (x, y) theoretical space will become a heavy plate with a nonuniform mass distribution given by the theoretical b-values, Figures 11A and 11B.

A set of contour plots of constant b-values visulizes the properties of the *b*(*x*, *y*) surfaces or, equivalently, the properties of the associated rigid bodies. If the input stress consists of two shocks of different temperatures but same duration, the theoretical constant contour plots look like in Figures 11A and 11B. Although they resemble each other, the contours for the durations *D*_1 _= *D*_2 _= 15 minutes are rotated counterclockwise with respect to those for which *D*_1 _= *D*_2 _= 20 minutes, Figure 11C.

The global property that we are looking for is the extent of this rotation. The rigid body perspective helps at this point because the principal axes of the plate gives the global orientation we are looking for [18]. The principal axes are perpendicular to each other and only one will be used to describe the global orientation.

The experimental angle between the principal axes confirms the theoretical prediction that the the contours for the durations *D*_1 _= *D*_2 _= 15 minutes are rotated counterclockwise with respect to those for which *D*_1 _= *D*_2 _= 20 minutes.

In addition to the estimated b-values from experiments, we also have their experimental 95% confidence bounds. It is worthwhile to check if the counterclockwise orientation is predominantly preserved if b-values, randomly sampled from their experimental 95% confidence bounds, are used. From these random samples we obtained that the probability of the counterclockwise rotation is 0.71, which is in line with the theoretical prediction.

**Figure 12:**
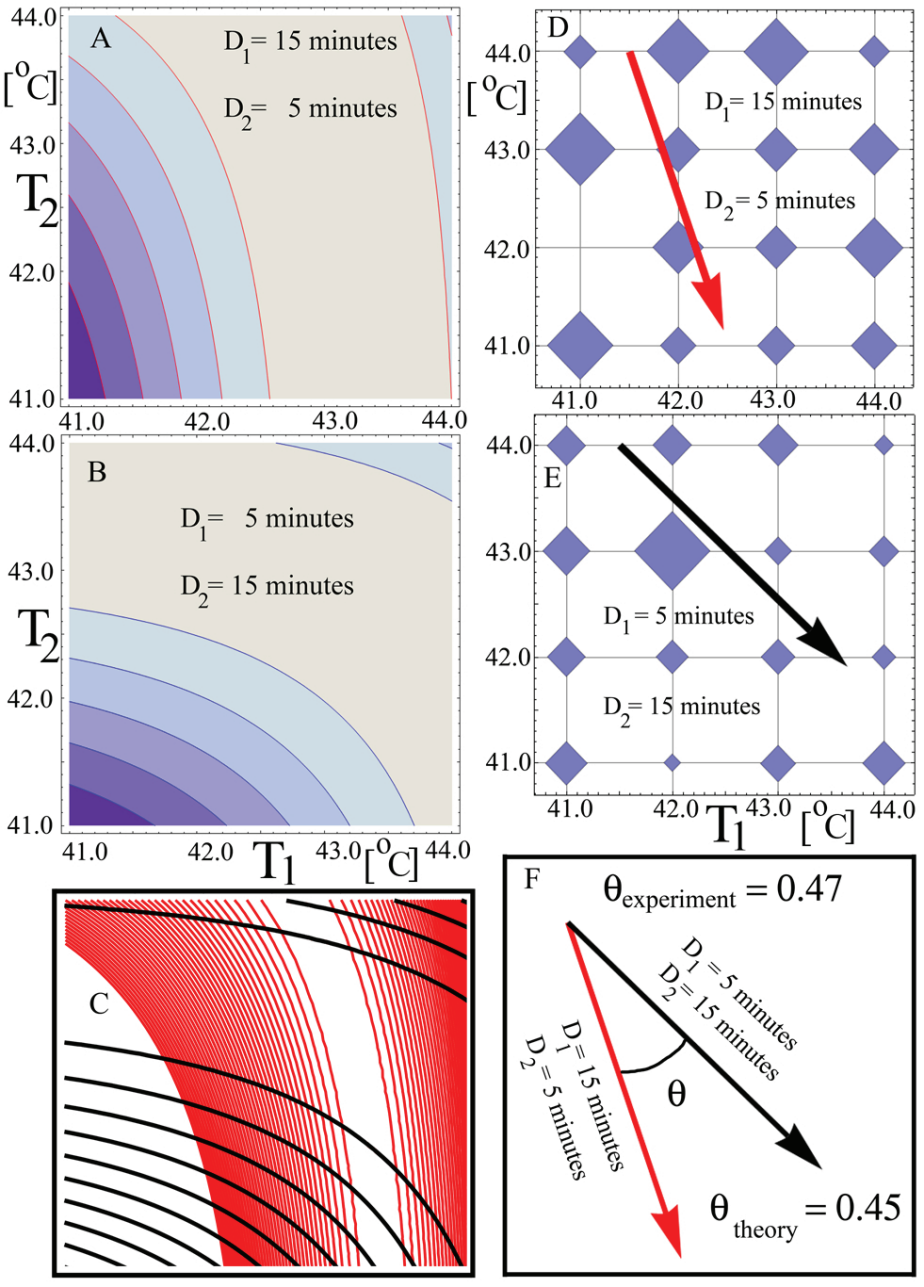
**The rotation angle increases for unequal shock durations**. The rotation of the contour plots (A) and (B) relative to one another increases because the ratio of the shock durations increases from 1 in, Figure 11, to 3 for this figure, (C). The principal axes shown in (D) and (E), obtained from the experimental data, encompass an angle that is close to the theoretical predicted angle (F).

Although the experimental results does not contradict the theory, we would like to see a rotation angle greater than *θ_experiment _*= 0.11 from Figure 11. An insight into designing further experiments comes from the theoretical model, which can be used to find such experimental conditions that increase the rotation angle. The model shows that the rotation angle increases if the durations of the shocks are significantly different. Theoretical results for unequal shocks' durations are shown in Figure 12. The specific durations of 5 and 15 minutes were chosen to maximize the ratio of the shocks' durations and to minimize experimental errors in recording the durations. The theoretical model also shows that, for these durations, the maximum b-value falls in the middle of the contour plots of Figure 12, which produces a detectable variation in the b-values across the temperature range 41°*C *to 44°*C*.

The theoretical rotation angle for Figure 12 is *θ*_*theory *_= 0.45. From two independent experiments in the same range of 41°*C *to 44°*C*. (Experiment 8 and 10 from Material and Methods), the experimental angles are *θ*_*Experiment*8 _= 0.48 and *θ*_*Experiment*10 _= 0.47 respectively, in good agreement with the predicted value. Figure 12 shows the results for Experiment 10.

The angle remains positive in a new set of experiments (Experiment 9 from Material and Methods) for which temperatures were dropped by 0.5°*C*, covering the range 40.5°*C *to 43.5°*C*. The experimental angle is *θ*_*Experiment*10 _= 0.12 whereas the theoretical one is *θ_theory _*= 0.34. It is obvious that intrinsic stochastic biological processes as well as experimental noise influence the numerical agreement between theoretical and experimental angles. However, in all four experiments analyzed above, the experimental angles are all positive, as the theory predicts.

To check further the positivity property of the rotation angle, we sample b-values randomly from their experimental 95% confidence bounds. The probability of the angle to be positive is 0.71, 1.00, 0.92, and 0.60 for Experiment 1 and 2, Experiment 8, Experiment 9, and Experiment 10 respectively. This is in good agreement with the theoretical prediction.

**Figure 13:**
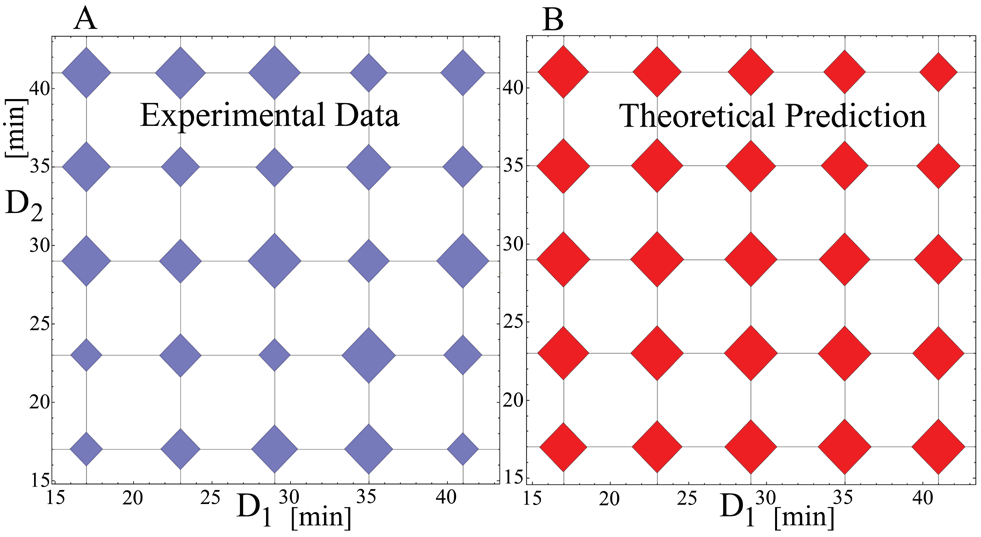
**Two shocks with variable durations and constant temperature**. Both the experimental data and theoretical predictions show a less variability among the promoter activities than for the case of variable temperature experiments.

In the two-shock experiments analyzed so far, the temperatures were chosen to cover a range of values, whereas the durations were kept the same. The opposite situation was also investigated. The durations of the shocks were varied through all 25 combinations of 17, 23, 29, 35 and 41 minutes whereas the the first shock was delivered at 40.5°*C*, and the second shock at 41.5°*C*, (Experiment 5 in Materials and Methods). We do not have an angle of rotation to compare *b*(*x*, *y*) surface for this experimental design. We will use instead a different global measure, namely the flatness of the surface, as is described below. The theoretical model for this experimental design predicts that the variation of the b-values over the entire range of durations is less than in the case of variable temperature range, compare Figure 13 with Figure 12. In other words, the surface *b*(*D*_1_, *D*_2_) is more at than the surface b(*T*_1_, *T*_2_), each considered on the corresponding experimental conditions. The variance of the set of discrete data points over the range of experimental conditions is used to measure the flatness. We find that the mean value of the variance of *b*(*T*_1_, *T*_2_), computed from Experiments 1, 2, 8, 9, and 10, is 4.26 times larger than the variance of *b*(*D*_1_, *D*_2_), computed from Experiment 5. On the other hand, the theoretical prediction is that the variances are in the ratio of 4.41, in agreement with the experimental value.

The Experiment 5 with variable durations was designed to avoid sever stress responses, so temperatures as low as 40.5°*C *and 41.5°*C *were used, which may explain the flatness. The severe stresses bend the *b*(*T*_1_, *T*_2_) surface and thus help to better distinguish different experimental conditions. This type of bending was employed in increasing the rotation angle which was studied above.

We end the analysis of the consistency between the experimental data and the theoretical model by looking into the robustness of the fitted parameters. The double-shock experiments were used to test the theory based on the parameter set *α *= 0.63°*C*^-1^, *β *= 0.026 min^-1^, , , and *m *= 0.67 obtained exclusively from the one-shock experiments. If the goal is to fit the model parameters to data, then it is better to pull together all experimental results. This may be done because a score can be computed for two-shocks also:(29)

**Figure 14:**
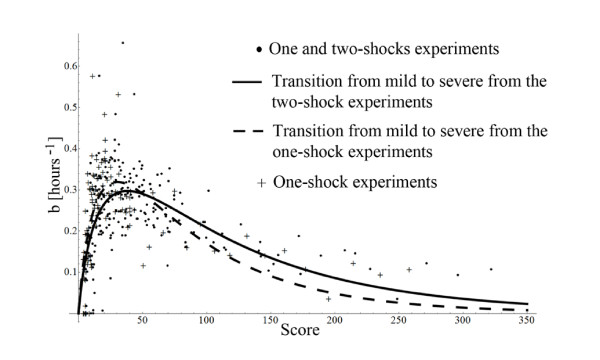
**The robustness of the theoretical model**. The parameters for all three blocks of the model in Figure 9 do not suffer a large change when more data is used for fitting.

Using the same procedure of finding first (*α*, *β*) and then (*A*, *λ*, *m*), we get *α *= 0.71°*C*^-1^, *β *= 0.023 min^-1^, ,  and *m *= 0.62. The comparison between the results, obtained using the one-shock scores only versus using one and two-shocks together, is presented in Figure 14. An encouraging result is that the new added points line on the tail of the transition curve without imposing a major deformation on it. Moreover, the global distribution of points in Figure 14 is not extensively altered when double-shock experiments are added. The same conclusion emerges when the parameters *α*, *β*, ,  and *m *are compared.

The NLN model can be used to predict the response to input heat stress that are not single or double shocks. In [3] a slow heating from 37°*C *to 42°*C *was considered with the result that the Hsp70 mRNA will accumulate at much lower levels than for a shock that brings the temperature immediately to 42°*C*. To check that the NLN cascade predicts a similar effect, we computed the b-value for three input profiles: (i) a shock for 0.5 hours (ii) a ramp 37 + 10*t *that reaches 42°*C *after 0.5 hours and (ii) a quadratic accumulation 37 + 5/(0.5)^2^*t *which reaches the same 42°*C *temperature after 0.5 hours. The slowest heating profile is (iii) whereas (i) is the fastest, that is (iii) < (ii) < (i). The computed b-values, at the end of the heat stress, are: 0.07 for (iii), 0.10 for (ii) and 0.24 for (i). Given that smaller b-values imply weaker transient activity, we find that a slow heating profile generates a weak transition activity. This conclusion is in agreement with the conclusion of [3].

We explained so far the connection between Input_1 _and Onput_3 _of Figure 9 and the networks of Figure 1. It is not the purpose of this study to reduce the network of Figure 14 to their equivalent system of Figure 1A or to find the optimal representation in terms of an NLN cascade of Figure 1B. It will be interesting though to compute the equivalent systems for all three networks of [2-4] and conduct a comparative study.

## Discussions and Conclusions

Most of the models in molecular biology are mechanistic. For example, the models may describe which proteins interact, what are the downstream effects or how molecules are transported between different compartments within the cell. The model that we described in this study is not mechanistic. For a biochemically oriented molecular biologists this approach may be unfamiliar. However, the systems-level approach in biology is not new [19,20]. With the present interest in systems biology, the system-level theoretical approach was revitalized [21,22]. Contrary to biology, the theories of modern engineering are heavily based on the systems-level design. The logic of block diagrams and signals processed by the blocks (modules) occupies a central place in control engineering, for example. For the heat shock system in bacteria, cellular block diagrams and functional modules were constructed and analyzed in [23]. The study [23] points to the fact that because biological networks are complex regulatory systems, it is useful to seek similarities between biological signal processing pathways and the functional modules traditionally identified in control engineering. This rationale was the starting point for this paper, where the functional cascade modules N, L and N are used to model the experimental data.

In control and dynamical systems theory the modular decomposition is used to make modeling and model reduction of systems more tractable, [23]. Model reduction is an old and useful procedure used to study the flow of information through systems. A simple but powerful example is the Thévenin reduction procedure for electric circuits. Its presentation from [24] is short and enlightening:" Thévenin theorem states that any two-terminal network of resistors and voltage sources is equivalent to a single resistor in series with a single voltage source. Any mess of batteries and resistors can be mimicked with one battery and one resistor". To translate its fundamental idea to biology, we may imagine that any mess of some specific molecular interactions can be mimicked with a few molecular interactions. If such a level of understanding is achieved in molecular biology, then synthetic biology will be the first to profit and we will be able to design close to optimal biological devices.

In model reduction we thus start from an extended network of interactions and proceed towards finding a few functional blocks that mimic the extended network for which an input and an output signal were identified. However, for the heat shock system the extended network is absent at present, and the only way to obtain a reduced model is to identify an input and an output signal and run experiments. We employed this procedure for the b parameter which measures the lifetime of the transient response. To summarize the results, as a compact mathematical formula, the lifetime of the transient response,(30)

is given by(31)

with *α *= 0.71°*C*^-1^, *β *= 0.023 min^-1^, *A *= 0.054 hour^-2^, *μ *= 0.00127 min^-1 ^and *m *= 0.62.

The above parameters were computed using the estimated values *α *= 0.71°*C*^-1^, *β *= 0.023 min^-1^, ,  and *m *= 0.62, obtained from all experimental data.

The parameter *a *from Transient(t) was not analyzed because its estimation accuracy from the available experimental data is less than the accuracy level obtained for *b*, which is based on precise time measurements. To create a model for *a *we need to increase the number of input heat shocks into thousands with the help of an automated robotic system. As we mention above, this leap in the number of inputs will show subtle structures in the *b *function, which are not captured by the present model, plus it will reveal the model for the parameter *a*.

The structure of the mathematical model (31) is represented by the NLN model from Figure 9.

The NLN structure is built on a series of general ideas related to thermotolerance without a recovery time, sharp sensitivity to small variations in temperature and the existence of mild and severe classes of responses to stress. The model is based on experimental data collected for a series of one or two adjacent heat shocks of variable durations and variable temperature. The durations were in the range of 5 to 50 minutes and temperatures from 40°*C *to 42°*C*. The cells were returned to 37°*C *at the end of the heat stress. The measurements were taken after the end of the heat shock. In contrast with our experimental approach, the majority of the experimental data used for the models [2-4] were taken by exposing the cells to a prolonged continuous heat shock [7,25] usually at 42°*C*. The samples were taken during the continuous heat shock. Beside the dominant continuous shock method, few one shock experiments were also considered. The study [3] used the experimental results of [25] for a one shock stress of 40 minutes at 40°*C*, 41°*C*, 42°*C*, 43°*C*. In [4] the cells were subjected also to a continuous heat shock at 42°*C *and samples were taken during the shock. Our mathematical model and those from [2-4] obviously depend on the experimental conditions considered. An encouraging aspect is that all the models predicts a transient response of the cell to a heat shock. In [2-4] the transient response was modeled through differential equations based a mechanistic network of molecular interactions whereas in our study we considered an input-output cascade model and concentrated on the life time of the transient response as a function of the heat shock input.

The theory will evolve as more data about the biological structure of the heat shock system will become available. Such a theory will contain input-out blocks that will separately explain the three main regulatory modules: transcriptional activity, mRNA stability and translational efficacy. However, the basic three general ideas related to thermotolerance without a recovery time, sharp sensitivity to small variations in temperature and the existence of mild and severe classes of responses to stress will still be present in the improved theory. Because biological systems are made of stochastic devices, the transient activity is best described by a stochastic theory. However, we cannot propose such a theory at this point because thousands of different stress inputs are needed to build such a theory. A stochastic theory for the GFP accumulation was achieved in [5] because we had access to thousands of measured cells through the flow cytometry technique.

Given the present trends in systems and synthetic biology, we are optimistic that an improved stochastic theory for the heat shock system is ultimately achievable.

## Methods

### The heat shocks

The construct used in this study was described in [5], as well as the heat shock procedures for one shock and GFP fluorescence intensity detection and analysis. In this study, more conditions (up to 32) were tested simultaneously plus two shocks were used as input signals. The cells were detached with trypsin and allowed to recover in suspension in complete growth medium for 3 to 4 h at 10^6 ^cells/mL at 37°*C *in a CO2 incubator. The cells were then aliquoted in 50 mL conical tubes, one for each experimental condition (temperature and duration of heat shock). A precision water-bath was used for each temperature. The temperature of each water-bath was accurately monitored with a precision Hg thermometer (accuracy ±0.1°*C*). Then the cells were centrifuged, the medium was aspirated, and the heat was initiated by resuspending the cell pellet quickly at 5 × 10^5 ^cells/mL in a medium prewarmed at the temperature selected for the heat shock. The tube was then placed in the same water-bath for the remainder of the heat shock, after which the tube was placed in ice-cold water and agitated for the amount of time that had previously been determined to be necessary to bring the temperature back to 37°*C *(the icing time was from 2 to 14 seconds). The tube containing the cells was then placed in the incubator set at 37°*C*. For experiments involving a second shock, after the temperature was brought back to 37°*C*, the cells were centrifuged again, the medium was aspirated, and the second heat shock was initiated by resuspending the cell pellet quickly in the medium prewarmed at the second shock's temperature. The processing time between shocks was kept short, for about 2 minutes. This procedure ensures that two distinct shocks were applied and that the time between shocks is very short, much shorter than the shocks' durations. From that point on, samples were taken every 2 h for up to 25 h. The cells were kept in suspension in the 50 mL tubes in a CO2 incubator at 37°*C *for the rest of the experiment.

In all experiments, a control where the cells were kept at 37°*C *for the whole time was included. The exact duration of each heat shock was monitored with a stopwatch and by a computerized voice coded using MATLAB (MathWorks). The voice control code was based on a detailed time-line design that was optimized to produce a minimal total time of cell stress manipulation, maximizing, at the same time, the accuracy and precision of the experiments. The voice control choreographed the movements of five persons during the input stress phase of the experiment. This protocol allowed a very strict control of the thermal shocks applied to the cells. The sampling procedure and flow cytometry data analysis were presented in [5].

### Method used to estimate the angle of rotation

The vectors that defined the global orientation of *b*(*T*_1_, *T*_2_) are the eigenvectors of the moment of inertia tensor. For a 2-dimensional discrete heavy structure, the moment of inertia tensor, in the coordinates defined by the experimental conditions, is a 2 by 2 matrix:(32)

where, for *i *= 1, 2 and *j *= 1, 2,(33)

Here *δ_ij _*= 1 if *i *= *j *and zero otherwise. The hat above the temperatures means that they are considered with respect to the center of mass of the discrete heavy plate:(34)

The statistical data analysis was performed with MATLAB (MathWorks) and the mathematical model with Mathematica(Wolfram). The description of experiments is presented below.

### Conditions used in the experimental design

**Experiment 1 **Two shocks, each one for 20 minutes. The temperatures were all 16 combinations of 41°*C*, 42°*C*, 43°*C *and 44°*C*. Also, we did one-shock four experiments of 20 minutes for each temperature in the list. Total conditions = 20.

**Experiment 2 **The same design as for the Experiment 1, but each shock had a 15 minutes duration. Total conditions = 20.

**Experiment 3 **Consists of two simultaneous temperature conditions. One shock at 41°*C *at durations, from 15 to 35 minutes in steps of 2 minutes. The other condition is one shock at 42°*C *for the same durations. Total conditions = 22.

**Experiment 4 **Consists of two simultaneous experiments. One shock at 40°*C *at durations, from 20 to 40 minutes in steps of 2 minutes (except for 36 minutes which was lost). The other condition is one shock at 41°*C *for the same durations. Total conditions = 21.

**Experiment 5 **Consists of two shocks of different durations. The first shock was delivered at 40.5°*C*, whereas the second shock at 41.5°*C*. The durations were all 25 combinations of 17, 23, 29, 35 and 41 minutes. Total conditions = 25.

**Experiment 6 **Consists of two simultaneous experiments. One shock at 40°*C *at durations, from 20 to 40 minutes in steps of 2 minutes. The other condition is one shock at 42°*C *for the same durations. Total conditions = 22.

**Experiment 7 **One shock at 39°*C *for duration from 20 minutes by 5 minutes to 125 minutes. Total conditions = 22.

**Experiment 8 **Consists of two shocks at different durations and different temperatures. In one set of conditions we delivered the first shock for 5 minutes followed by a second shock of 15 minutes. The temperature pairs were all combinations of 41°*C*, 42°*C*, 43°*C *and 44°*C *except the diagonal combinations (41°*C*, 41°*C*), (42°*C*, 42°*C*), (43°*C*, 43°*C*). For the second of conditions, we delivered the first shock for 15 minutes and the second shock for 5 minutes at the same temperature pairs. Total conditions = 24.

**Experiment 9 **The same design as for Experiment 8 but at temperatures 40.5°*C*, 41.5°*C*, 42.5°*C *and 43.5°*C*. Total conditions = 24.

**Experiment 10 **The same design as for Experiment 8 but including the diagonal conditions (41°*C*, 41°*C*), (42°*C*, 42°*C*), (43°*C*, 43°*C*). Total Samples = 32.

**Experiment 11 **Consists of one shock at 43°*C *for durations from 6 to 75 minutes in steps of 3 minutes. Total Samples = 24.

**Experiment 12 **One shock for 20 minutes at temperatures 40.5°*C*, 41.5°*C*, 42.5°*C *and 43.5°*C*. from different cell batches. Total Samples = 16.

## Authors' contributions

OL designed the study and wrote the report. All authors contributed to the experimental work and data analysis. All authors read and approved the final manuscript.
